# Editorial: Recent advances and challenges in cancer immunotherapies for patients with autoimmune diseases

**DOI:** 10.3389/fimmu.2022.1127739

**Published:** 2023-01-09

**Authors:** Reem Saleh

**Affiliations:** ^1^ Peter MacCallum Cancer Centre, Melbourne, VIC, Australia; ^2^ Sir Peter MacCallum Department of Oncology, The University of Melbourne, Parkville, VIC, Australia

**Keywords:** cancer, immune checkpoints, case reports, predictive biomarkers, personalized medicine, autoimmune disease

This Research Topic gathers different contributions (case reports, research articles and review articles) describing immune related adverse events in cancer patients with preexisting autoimmune diseases or exposed to immunotherapy. Furthermore, the topic discusses new approaches to discover new biomarkers that predict the development of adverse events post immunotherapy.

The first article of this Topic (Zhou et al.), introduces a case report describing autoimmune encephalitis (AIE), for the first time, as a neurological immune-related adverse event (n-irAE) of toripalimab (a new immune checkpoint inhibitor targeting PD-1) in metastatic melanoma patient. This has revealed the safety profile of this new immune checkpoint inhibitor and further studies are required to validate this finding in other cancer types.

The second article by Tang et al. describes a retrospective study in cancer patients with pre-existing autoantibodies treated with anti-PD-1 therapy. Authors reported the association between the presence of autoantibodies and overall survival in patients. Authors concluded that PD-1/PD-L1 inhibitors could be administered safely and effectively in patients with preexisting autoantibodies but without active autoimmune disease. However, patients positive for antithyroid antibody should monitor closely thyroid dysfunction during anti-PD-1/PD-L1 treatment.

Computational approaches to optimize patient selection for immunotherapy in breast cancer patients were described in Jiao et al.. This would particularly help in improving personalized medicine and avoid unnecessary irAEs.

Comprehensive review articles by Tang et al. and Zhou et al. shed the light on anti-PD-1/PD-1 therapy and novel immune checkpoint inhibitor, anti-VSIG and the associated risk of developing irAEs in cancer patients following treatment. These articles emphasized the importance of finding new biomarkers that predict the risk of developing irAEs to optimize future application in cancer patients.

The connection between the development of autoimmune reactions and immune checkpoint inhibitors has been investigated in Mari et al.. Authors describe a case report of a refractory bullous pemphigoid (BP) which occurred four years after nivolumab introduction and lasted despite nivolumab discontinuation in a patient whose metastatic renal carcinoma is still controlled after more than two years without any anticancer treatment. This may highlight the potential association between irAE and response to immune checkpoint inhibitors. This underlines the existence of late-onset and long-lasting irAEs even after discontinuation of treatment, which should encourage clinicians to remain vigilant over the long term.

Haas et al. proposed a new immune checkpoint receptor, Siglec-7, which its expression could improve CD8+ T cell anti-tumour immune response and potentially improve approaches for personalized medicine and for T cell-driven autoimmune diseases and cancer.

The efficacy of anti-PD-L/anti-CTLA-4 immunotherapy in lung cancer patients and the potential of developing irAEs were assessed in Dey et al.. Authors found that the efficacy of immunotherapy was improved in patients who developed on-treatment irAEs. This was independent of severity of iAEs or the need for steroid treatment, which is important in allowing patients to remain on treatment and derive optimal clinical benefit. Further research is warranted to establish the correlation between incidence of irAEs and efficacy in this patient population.

Alternatively, a computational approach by Glehr et al. concluded that finding predictive biomarkers for irAEs in cancer patients treated with immunotherapy is challenging and requires further studies.

We hope that the reader will find in this Research Topic a useful reference for the connection between autoimmune diseases and the application of immunotherapy in cancer patients, and recent approaches to find new predictive biomarkers to reduce the risk of irAE development and help in optimization therapy selection for patients.

## Author contributions

The author confirms being the sole contributor of this work and has approved it for publication.

